# Associations of galectin-3 expression and LGALS-3 (rs4652) gene variant with coronary artery disease risk in diabetics

**DOI:** 10.5937/jomb0-30424

**Published:** 2021-09-03

**Authors:** Basma A. Ibrahim, Samy H. Mohamed, Mohamed M.M. Hassaan, Norhan A. Sabbah

**Affiliations:** 1 University of Zagazig, Faculty of Medicine, Medical Biochemistry Department, Zagazig, Egypt; 2 University of Zagazig, Faculty of Medicine, Internal Medicine Department, Zagazig, Egypt

**Keywords:** LGALS-3 (rs4652), galectin-3 expression, IL-6, T2DM, CAD, LGALS-3 (rs4652), galectin-3 expression, IL-6, T2DM, CAD

## Abstract

**Background:**

Galectin-3 protein encoded by lectin galactoside-binding soluble-3 (LGALS-3) gene is an important genetic factor in type 2 diabetes mellitus (T2DM) and its cardiovascular obstacles in various populations. We aimed to elicit the pro-inflammatory effect of galectin-3 as determined by interleukin-6 (IL-6) serum levels and to explore the relationship between galectin-3 (LGALS-3 rs4652) gene variant and its expression levels with coronary artery disease (CAD) risk among T2DM Egyptian patients.

**Methods:**

112 lean subjects were compared to 100 T2DM without CAD and 84 T2DM with CAD. A tetra-primer amplification refractory mutation system polymerase chain reaction was used to test LGALS-3 (rs4652), and galectin3 expression was tested with a quantitative real-time polymerase chain reaction. Serum IL-6 was measured using an enzyme-linked immunosorbent assay.

**Results:**

We found that the prevalence of LGALS-3 (rs4652) AC genotype and galectin-3 gene expression levels in T2DM with CAD were significantly higher than the additional 2 groups and were correlated positively to IL-6 circulating levels. Also, the C allele carriers (AC+CC) had significantly higher relative Galectin-3 expression levels compared to the A allele carriers (AA).

**Conclusions:**

We concluded that galectin-3 expression levels and LGALS-3 (rs4652) AC genotype were coronary artery disease risk factors in people with type two diabetes among an Egyptian sample.

## Abstract (sr)

**Uvod:**

Protein galektin-3 kodiran genom LGALS-3 je važan genetski faktor kod dijabetes melitusa tipa 2 (T2DM) i pridruženim kardiovaskularnim problemima kod različitih populacija. Cilj NAM je bio da izazovemo pro-inflamatorni efekat galektina-3 određenim u zavisnosti od nivoa interleukina-6 (IL-6) u serumu, i istražimo vezu između varijante gena galektin-3 (LGALS-3 rs4652) i nivoa njegove ekspresije sa bolešću koronarne arterije (CAD) kod pacijenata iz Egipta sa T2DM.

**Metode:**

Uporedili smo 112 mršavih ispitanika sa 100 ispitanika koji imaju T2DM bez CAD i 84 sa T2DM sa CAD. Za ispitivanje LGALS-3 (rs4652) korišćena je amplifikovana lančana reakcija polimerazne refraktornog mutacionog sistema sa tetra-prajmerom a ekspresija galektin-3 testirana je kvantitativnom lančanom reakcijom polimeraze u realnom vremenu. Serum IL-6 je meren korišćenjem enzimski povezanog imunosorbentnog testa.

**Rezultati:**

Otkrili smo da su prevalencija nivoa genotipa LGALS-3 (rs4652) AC i nivoa ekspresije gena galektin-3 kod pacijenata sa T2DM sa CAD bili znatno veći od dodatne 2 grupe i pozitivno povezani sa nivoima cirkulacije IL6. Takođe, nosioci alela C (AC + CC) imali su značajno veće relativne nivoe ekspresije galektina-3 u poređenju sa nosiocima alela A (AA).

**Zaključak:**

Zaključili smo da su nivoi ekspresije galektina-3 i genotip LGALS-3 (rs4652) AC faktori rizika od bolesti koronarne arterije kod ljudi sa dijabetesom tipa dva na uzorku iz Egipta.

## Introduction

Diabetes mellitus (DM) is a major health problem and has reached unprecedented rates. Nearly half a billion people worldwide live with diabetes in 2019 (9.3 per cent of adults aged 20-79 years). DM is a rapidly growing health issue in Egypt; the number of diabetics in 2019 was 9.8 million and is expected to be 11.9 million by 2030 and 16.9 million by 2045 [1]. The prevalence of type 2 diabetes mellitus (T2DM) in Egypt is around 15.6 per cent of all adults aged 20 to 79, with 86,478 DM-related deaths per year [2].

T2DM is a significant cardiovascular disease (CVD) risk factor, known to be the leading death cause among T2DM patients; at least 60% of DM patients die from CVD [3]. Atherosclerosis, the primary cause of coronary artery disease (CAD), is a more prevalent macro-vascular complication of atherosclerotic processes in T2DM, with increasing the duration of DM [4].

In such patients, cardiovascular risk assessment is critical because it affects decisions about the duration of follow-up and the testing methods used. Genetic tests are helpful to determine predictive factors for CVD diagnosis and early detection in DM patients.

Galectin-3 (*Gal-3*) is a member of the -galactoside-binding family of proteins [5]. It is produced by numerous cells, including vascular, epithelial, interstitial, and immune cells. It is present in both intracellular and extracellular spaces. Extracellular Gal-3 reacts with galactoside remains of extracellular matrix and cell surface glycoproteins through its C-terminal carbohydrate-recognition part, while intracellular Gal-3 reacts peptide-peptide interactions through its N terminal part [6] [7].

Such structure and position properties have identified that *Gal-3* takes part in several biological processes, including oxidative stress, apoptosis, vascular lesion, inflammation, and the progress of insulin resistance that may affect cardiovascular function and progression of chronic hyperglycemia [8].

*Gal-3* acts as an advanced glycation end products receptor, thus *Gal-3*'s position on the emergence and advancement of long-term DM complications by its ability to bind advanced glycemic end products (AGEs) to advanced lip-oxidation end products (ALEs) which build up in the target organ and influence its toxic effects by causing pro-inflammatory and prooxidising pathways [9].

The binding of AGEs with *Gal-3* on the cell surface forming a protein complex, then the adhesion between VSMC and matrix glycoprotein is weakened. This induces VSMC proliferation and migration to intensify atherosclerosis [10].

Also, *Gal-3* is a key regulator of essential pathways for acute and chronic inflammatory disorders; it can stimulate the release of pro-inflammatory cytokines such as tumour necrosis factor-α (TNF-α) and IL-6 via macrophage activation in a dose-dependent method [11].

*Gal-3* is a 32-to 35kD glycoprotein coded in chromosome 14 (14q22.3) by lectin galactoside binding soluble three genes (*LGALS-3*) and made up of 6 exons and five introns [12]. Various variants of single nucleotides in* LGALS-3* are found in exon 3 of chromosome 14 and influence its gene expression, such as; rs2274273, rs4644, and rs4652 [13].

It is the best candidate for genetic studies because of the function of the *LGALS-3* gene in the pathogenicity of different inflammatory conditions like DM.

Therefore, the purpose of this research was to define the potential association of *LGALS-3* rs4652 A/C genetic variant with gene expression with the risk of CAD in T2DM among the Egyptian population.

We evaluated *LGALS-3* rs4652 A/C genetic variant and its gene expression in type two diabetic patients and type two diabetic patients with CAD comparing them to a group of lean controls for achieving our aim.

### Subjects and methods/ Subject selection

This research was conducted in the departments of Medical Biochemistry and Internal Medicine, Faculty of Medicine, Zagazig University. One hundred diabetics type two with no coronary artery disease and 84 diabetics type two with coronary artery disease were involved in the Zagazig University Hospitals case-control study and compared to one hundred and twelve lean subjects.

EpiTools Epidemiologic calculators measured the sample size to estimate the statistical efficiency, showing a potential of at least 87.1 per cent [14].

The exclusion criteria were liver diseases, respiratory diseases, kidney dysfunction, inflammatory disorders lasting for 1 month, patients who take any lipid-lowering medications, family history of hyperlipidemia, patients on anti-inflammatory drugs like NSAID or corticosteroids, valvular heart disease, aortic aneurysms, heart failure, malignant tumours, type 1 DM, other metabolic disorders except for T2DM, or additional diseases which may affect the test results not considered as subjects of the analysis as well as patients who refuse to give consent and expressed lack of cooperation. CAD patients without appropriate basic details were also omitted.

All subjects were Egyptian, the control group undergoing a routine examination, all free from any history of obesity, high blood pressure, hyperlipidemia, DM, or CAD. The diagnosis of CAD was established by the 1978 World Health Organization (WHO) guidelines and/or 50% luminal stenosis via angiography in at least one main coronary artery. The WHO guidelines for diagnosis: (1) a history of at least 30 minutes of ischemic chest pain; (2) characteristic electrocardiography (ECG) changes; and (3) dynamic variations in the levels of myocardial enzymes cTnT or cTnI. Type 2 DM was diagnosed if fasting plasma glucose level ≥6.993 mmol/L, 2-h 75 g OGTT glucose level ≥11.1 mmol/L or random plasma glucose level ≥11.1 mmol/L, or if they were receiving glucose-lowering medications with an actual diagnosis [15]. We measured the heights and weights of the study subjects, and BMI was estimated as the body weight in kilograms divided by the height square in meters (kg/m^2^). The data on the subjects' smoking habits was obtained. Hypertension was diagnosed if systolic blood pressure >140 mmHg and/or diastolic blood pressure >90 mmHg or if the patient was consuming antihypertensive treatments [16]. Hyperlipidaemia was defined as total serum cholesterol levels >13.32 mmol/L, low-density lipoprotein cholesterol (LDL-c) >7.215 mmol/L, or serum triglycerides >9.99 mmol/L, or if the patient takes lipid-lowering drugs [17]. All participants were granted verbal plus informed written consent, with the research being accepted by the Faculty of Medicine's Ethics Review Committee, University of Zagazig. The study of research involving humans was performed following the Code of Ethics of the World Medical Association (Helsinki Declaration).

### Biochemical analysis

After skin sterilisation with ethyl alcohol swabs, seven ml samples of blood were taken from all subjects involved in the study. Three ml was taken on Ethylene-diamine tetra-acetic acid having tubes for DNA extraction, messenger RNA extraction, and glycated hemoglobin (HbA1c) estimation. Three ml was left and centrifuged for 15 minutes at 3000 rpm, and samples of serum were collected and stored at -20°C until analysed. The last 1 mL was put in tubes coated with sodium fluoride; then, the plasma was separated after centrifugation for blood glucose estimation.

Serum human IL-6 levels were measured using a new and optimised enzyme-linked immunosorbent assay kit (Human IL-6 ELISA kit, Bioneovan. Co. Ltd Beijing, China). Standardisation of the analysis was achieved according to the manufacturer's references, and results were normalised to a standard curve.

Routine enzyme methods (Spinreact) were used to measure fasting plasma glucose [18], total cholesterol (TC) [19], and triglycerides (TGs) [20]. HDL-c was calculated following apo-B-containing lipoproteins precipitation [21]. LDL-c was determined using the Friedewald formula [22]. We have evaluated the colourimetric levels of HbA1c using Biosystems (Barcelona, Spain) [23].

### DNA Extraction and polymerase chain reaction (PCR)

Genome DNA has been removed from EDTA anti-coagulated peripheral blood leucocytes following the instructions of the manufacturer using G-spin™ Total DNA Extraction Mini Kit provided by iNtRON Biotechnology, Korea. For further use, extracted DNA was preserved at -20°C. The purity of DNA could be detected by measuring A260 (the absorbance at 260 nm) to the A280 ratio. The pure double-stranded DNA sample is assumed to have an A260/A280 ratio varied from 1.7 to 1.9 [24].

### Detection of LGALS-3 gene variant

A tetra-primer amplification refractory mutation system polymerase chain reaction (T-ARMS PCR) was used to find *LGALS-3* gene variant. Two outer primers (forward outer, 5'-GGC TTA TCC TGG ACA GGC ACC TC-3' and reverse outer, 5'-TTT TTG ACT CTA CCA ACA TAC ACC CAT-3') as popular PCR reaction control primer and the two inner primers (forward inner, 5'-CAT CTT CTG GAC AGC CAA GTG TCA-3' unique for A allele and reverse inner, 5'-AGT GGC AGG GTA GGC TCC AGG-3' unique for C allele) have been created and used.

### Components of the 25 µL Rxn PCR reaction mixture

10 µL of 2X PCR Master mix Solution (i-Taq™), 5 µL of template DNA, 1 µL of every inner forward and reverse primer, 1.5 µL of every outer forward and reverse primer, and 5 µL DNase free water has been added into a 0.25 mL PCR tube.

### PCR Conditioning

PCR included cycles of denaturation of 95°C for 5 minutes then 30 cycles of denaturation at 95°C for 30 seconds, annealing at 67°C for 40 seconds, and extension at 72°C for 30 seconds with the final extension phase was at 72°C for 10 minutes.

**Figure 1:**
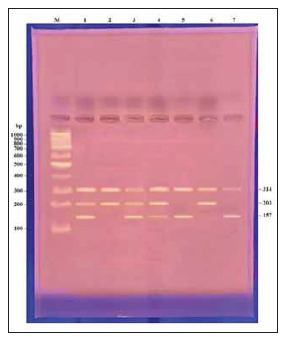
Agarose gel electrophoresis picture stained with ethidium bromide showing the PCR product in which there is the analysis of the Galectin-3 (LGALS-3 rs4652) gene polymorphism. DNA size marker (100bp) ladder. Lanes (1, 3, 4): AC genotype showing the presence of three bands 314+203+157 bp. Lanes (2, 6): AA genotype showing the presence of two bands 314+203 bp. Lanes (5, 7): CC genotype showing the presence of two bands 314+157 bp.

AG Eppendorf. Inc. Master-cycler, Hamburg, Germany, was used for the amplification reaction. The products of PCR were subjected to two per cent agarose gel electrophoresis, marked with ethidium bromide, and visualised by UV trans-illuminator. The product sizes for the A allele were 203 bp, for the C allele 157 bp, and 314 bp for the external control band (Figure 1).

### Messenger RNA extraction and quantitative real-time PCR (Q-PCR) of Galectin-3

Total RNA was isolated from the blood via easy-RED™ Total RNA Extraction Kit from iNtRON Biotechnology. Complementary DNA (cDNA) was reverse transcribed using HiSenScript™ RH (-) cDNA Synthesis Kit. Quantitative real-time PCR was performed: 10 µL of TOPreal™ qPCR 2X PreMIX (SYBR Green with low ROX), 1 µL of each forward and reverse primers, 2 µL of cDNA, and 6 µL of RNAase free water in 20 µL final volume. *GAPDH* gene expression was used as an internal control. 40 cycles; initial denaturation at 95°C for 15 minutes, followed by 95°C for 30 seconds, 53°C for 1 minute, and 72°C for 1 minute. The thermal program for the replication of both genes done under the same conditions containing, The primers for *Gal-3* forward: 5' CAG AAT TGC TTT AGA TTT CCA A 3 and Gal-3 reverse: 3 TTA TCC AGC TTT GTA TTG CAA 5,* GAPDH* forward: 5' ATG GAG AAG GCT GGG GCT 3' and *GAPDH* reverse: 3' ATC TTG AGG CTG TTG TCA TAC TTC TC 5'.

*Gal-3* gene expression levels were normalised to GAPDH as a housekeeping gene. Calculation and determination of the levels were performed using the CT system threshold process (2^-ΔΔCT^ method) [25].

### Statistical analysis

The data have been analysed with SPSS 22.0 for windows (SPSS Inc., Chicago, IL, USA) and MedCalc 13 for windows (MedCalc Software, Ostend, Belgium). Continuous variables were represented as mean ± standard deviation and range. Continuous variables were tested for normality by the Shapiro-Wilk test (sig) and Q-Q plot. ANOVA (F) test was used to compare the normally distributed data from three independent groups. Kruskal-Wallis test was used to compare three independent groups for non-normally distributed data. A Chi-Square test was used to compare three groups for qualitative data. All tests were two-sided p < 0.05 was considered statistically significant (S), p < 0.001 was considered highly statistically significant (HS), and p 0.05 was non statistically significant (NS).

## Results

The studied subjects comprised 47.97% males and 52.03% females. The three groups were almost age and gender-matched with no statistically significant difference, also smoking and BMI were not statistically significant differences (Table 1).

**Table 1:** Comparison of socio-demographic characteristics among the studied groups. 1^st^ group vs 2^nd^ group, (2) 1^st^ group vs 3^rd^ group, and (3) 2^nd^ group vs 3^rd^ group, ^^^=p-value for ANOVA test, ^^^^=p-value for Chi-square test, LSD = least significance difference. BMI, body mass index.

Variable	Group 1 Control (112)	Group 2 T2DM without CAD (100)	Group 3 T2DM with CAD (84)	P^^^	LSD
Age mean ± SD Range (years)	55.9±4.2 (45–62)	54.9±4.1 (45–62)	57.4±3.3 (50–62)	0.07	0.3(1) 0.2(2) 0.2(3)
BMI mean ± SD Range (kg/m^2^)	22.1±1.8 (19–25)	22.3±1.6 (19–25)	22.2±1.7 (20–25)	0.07	0.8(1) 0.6(2) 0.7(3)
Gender: Male (142) Female (154)	NO. (%)	NO. (%)	NO. (%)	P^^^^	
	56 (50.0%) 56 (50.0%)	44 (44.0%) 56 (56.0%)	42 (50.0%) 42 (50.0%)	1	
Smoking Yes (67) No (229)	20 (17.9%) 92 (82.1%)	26 (26.0%) 74 (74.0%)	21 (25.0%) 63 (75.0%)	0.6	

TC, LDL-c, TGs, HbA1c, FBG, PPBG, SBP, and DBP were statistically significantly higher among type two diabetics with CAD than people with type two diabetes with no CAD than the control group (p-value 0.001**) while HDL-c was statistically significantly lower among people with type two diabetes with CAD than people with type two diabetes with no CAD than the control group (p-value 0.001**) (Table 2).

**Table 2:** Comparison of the three groups based on laboratory data. 1^st^ group vs 2^nd^ group, (2) 1^st^ group vs 3^rd^ group, and (3) 2^nd^ group vs 3^rd^ group, *Statistically significant difference (P≤0.05), **Statistically highly significant difference (P≤0.001), ^^^=p-value for ANOVA test, LSD = least significance difference. TC, total cholesterol; LDL-c, low-density lipoprotein cholesterol; HDL-c, high-density lipoprotein cholesterol; TGs, triglycerides; HbA1c, glycosylated hemoglobin; FBG, fasting blood glucose; PPBG, 2-hour postprandial blood glucose; SBP, systolic blood pressure; DBP, diastolic blood pressure.

Variable	Group 1 Control (112) mean ± SD Range	Group 2 T2DM without CAD (100) mean ± SD Range	Group 3 T2DM with CAD (84) mean ± SD Range	P^^^	LSD
TC (mmol/L)	7.5869±1.1267 (4.995–9.213)	12.6596±0.9158 (11.1–14.2635)	15.7565±1.2821 (12.321–17.2605)	0.001**	0.001**(1) 0.001**(2) 0.001**(3)
LDL-c (mmol/L)	5.2004±1.0601 (3.33–7.3815)	9.6404±0.7437 (6.771–10.434)	10.928±0.9546 (9.435–13.542)	0.001**	0.001**(1) 0.001**(2) 0.001**(3)
HDL-c (mmol/L)	3.8517±0.5994 (2.7195–4.884)	2.1923±0.494 (1.665–3.663)	1.6262±0.3219 (1.221–2.997)	0.001**	0.001**(1) 0.001**(2) 0.001**(3)
TGs (mmol/L)	5.5722±1.1433 (3.774–7.77)	9.3185±0.9102 (5.55–9.99)	10.4118±0.4496 (9.213–11.0445)	0.001**	0.001**(1) 0.001**(2) 0.001**(3)
HbA1c (%)	4.6±0.4 (4–5.2)	6.6±0.8 (5.1–8.3)	8.9±0.8 (7.4–10.5)	0.001**	0.001**(1) 0.001**(2) 0.001**(3)
FBG (mmol/L)	5.2503±0.605 (4.107–6.2715)	9.9179±0.8825 (8.1585–11.1)	11.5107 ±0.7992 (9.99–13.2645)	0.001**	0.001**(1) 0.001**(2) 0.001**(3)
PPBG (mmol/L)	6.0384±0.7826 (4.551–7.77)	14.0915±2.3532 (9.435–18.315)	19.7414±3.1968 (11.1555–23.865)	0.001**	0.001**(1) 0.001**(2) 0.001**(3)
SBP (mmHg)	116.1±8.7 (100–130)	138.6±9.7 (120–150)	145.1±10 (130–160)	0.001**	0.001**(1) 0.001**(2) 0.01*(3)
DBP (mmHg)	77.5±6.4 (70–90)	88.9±6.8 (80–100)	95.1±10 (80–110)	0.001**	0.001**(1) 0.001**(2) 0.005*(3)

Galectin-3 expression was statistically significantly higher among type two diabetics with CAD than type two diabetics with no CAD than the control group (3.3±2.5 > 1.99±0.8 > 1.06±0.7, p-value 0.001**), the highest (CC genotype) percentage was among type 2 diabetes with CAD (62.5% versus 35.0% and 2.5%) of type two diabetes with no CAD and control groups, respectively. About half (50.5%) of A allele was among the control group versus (31.4% and 18.1%) of the type two diabetes with no CAD and type two diabetes with CAD groups respectively (p-value 0.001**) while most of the C allele (45.9%) was among type two diabetes with CAD group (Table 3).

**Table 3:** Comparisons of Galectin-3 expression, IL-6 levels, genotyping, and alleles in the studied groups. 1^st^ group vs 2^nd^ group, (2) 1^st^ group vs 3^rd^ group, and (3) 2^nd^ group vs 3^rd^ group, **Statistically highly significant difference (P 0.001), #=p-value for Kruskal-Wallis test, LSD = least significance difference IL-6, interleukin-6

Variable	Group 1 Control (112)	Group 2 T2DM without CAD (100)	Group 3 T2DM with CAD (84)	P#	LSD
Galectin-3 expression	1.06±0.7 (0.23–2.75)	1.99±0.8 (0.86-3.94)	3.3±2.5 (0.65–8.34)	0.001**	0.001**(1) 0.001**(2) 0.7(3)
IL-6 (pg/mL)	1.65±0.34 (1.29–2.89)	6.47±1.29 (4.18–8.89)	9.34±2.05 (7.21–11.74)	0.001**	0.001**(1) 0.001**(2) 0.001**(3)
Galectin-3 genotyping AA (116) AC (140) CC (40)	NO. (%)	NO. (%)	NO. (%)	P^^^^	
	77 (66.4%) 34 (24.3%) 1 (2.5%)	31 (26.7%) 51 (36.4%) 14 (35.0%)	8 (6.9%) 55 (39.3%) 25 (62.5%)	<0.001**	
Galectin-3 alleles A (372) C (220)	188 (50.5%) 36 (16.4%)	117 (31.4%) 83 (37.7%)	67(18.1%) 101 (45.9%)	<0.001**	

**Figure 2:**
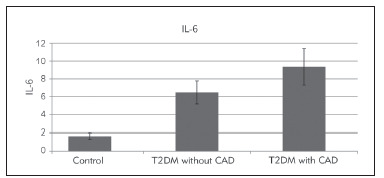
Bar chart for comparing IL-6 among the three studied groups.

**Figure 3:**
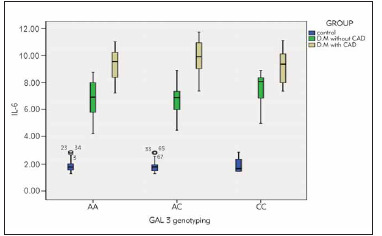
Box plot chart shows higher IL-6 among the T2DM with CAD group than the T2DM without CAD group than the control group. It also shows higher IL-6 among Galectin-3 genotype CC than genotype AC than genotype AA in each of the three studied groups.

IL-6 was statistically significantly higher among type two diabetics with CAD than people with type two diabetes without CAD than the control group (9.34±2.05 > 6.47±1.29 > 1.65±0.34 respectively, p-value 0.001**) (Table 3) and (Figure 2), also it was statistically significantly higher among *LGALS-3* genotype CC than AC than AA on each group, pvalue 0.001**) (Figure 3).

**Figure 4:**
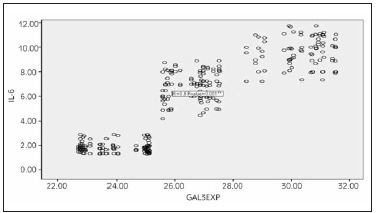
Scatter plot graph for the positive correlation between IL-6 and Galectin-3 expression among the three studied groups.

Galectin-3 expression was statistically significant positively correlated with IL-6 (r=0.8, P-value= 0.001**) (Figure 4).

**Figure 5:**
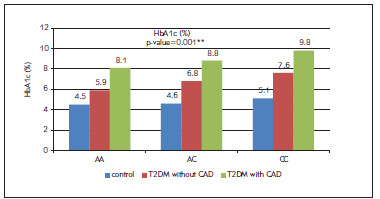
Bar chart for comparing HbA1c between the different Galectin-3 genotyping among the three studied groups.

On comparing HbA1c between the different Galectin-3 genotyping among the three studied groups, it was statistically significantly higher among type two diabetes with CAD than type two diabetes with no CAD than the control group and also statistically significantly higher among CC than AC than AA genotype on type two diabetes with and without CAD but not statistically significant differences among different genotypes of the control group (Figure 5).

**Figure 6:**
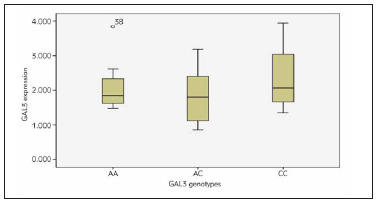
Box plot chart for the relationship between the Galectin-3 genotyping and its expression among the T2DM without CAD group.

**Figure 7:**
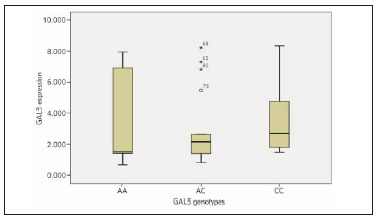
Box plot chart for the relationship between the Galectin-3 genotyping and its expression among the T2DM with CAD group.

There was no statistically significant difference in Galectin-3 expression between the different Galectin-3 genotyping among T2DM with and without CAD (Figure 6 and Figure 7).

## Discussion

Diabetes mellitus type 2 has been one of the world's most significant and most severe death epidemics in the last century. By contrast with non-diabetes, cardiovascular disease deaths in those with type 2 DM are higher. The most common cardiovascular complication is coronary heart disease in those with T2DM [4].

Genetic variants were also a new area in an epidemiological study to determine the genetic component underlying these risk factors and to combine T2DM with CVD.

The possible relationship between Gal-3 (LGALS-3 rs4652) gene variant and its expression with CAD risk in T2DM was studied in this study. To our knowledge, this is the first research in an Egyptian community that explores this relationship.

*Gal-3* is a carbohydrate-bound protein and has a vital role in the control of the inflammatory process. Its expression increased in human atherosclerotic lesions as it was involved in proliferation, macrophage chemotaxis, phagocytosis, neutrophil extravasation, oxidative stress, apoptosis, and vascular lesion. These mechanisms were implicated in cardiovascular diseases among T2DM patients [26].

Fibroblasts are the main cells in the process of tissue fibro-genesis; they can stimulate inflammation via secretion of multiple pro-inflammatory cytokines like TNF-α and IL-6, and chemokines such as C-C chemokine ligand 2 (CCL2), C-C chemokine ligand 3 (CCL3), C-C chemokine ligand 5 (CCL5), and C-X-C motif chemokine 8 (CXCL8), upon increased expression levels of *Gal-3* [27].

All of that trigger inflammation which is the main factor of atherosclerosis in T2DM patients and is considered a risk factor for vascular complications, like CAD, heart failure, peripheral artery disease, and other vascular complications [3].

IL-6 is a pro-inflammatory pleiotropic cytokine with a wide variety of cellular and humoral immune consequences linked to inflammation, immune response, and tissue damage [28]. IL-6 release is considered to play a crucial role in systemic inflammatory reactions and can be used to evaluate the body's inflammatory response [29].

In this research, the results have shown that the frequency of AA genotype of *LGALS-3* rs4652 gene variant was most common among the control group than T2DM without CAD and T2DM with CAD (66.4% > 26.7% > 6.9%), respectively, and the opposite was found in Gal-3 AC and CC genotyping which were higher among T2DM with CAD than T2DM without CAD than the control group (39.3% and 62.5% > 36.4% and 35.0% > 24.3% and 2.5%) respectively. Our results have shown that the AC genotype prevalence was significantly greater in T2DM with CAD patients than in the other two groups, which means that the rs4652 AC genotype was a CAD risk factor in Egypt's T2DM patients.

Also, we found that Gal-3 gene expression levels in T2DM with CAD patients were significantly higher than the additional two groups, and the genotypes C allele carrier (AC+CC, n=220) had associated *LGALS-3* mRNA expression significantly higher than the A allele carrier (AA).

Our findings go hand in hand with De Boer et al. [30] and Atabaki et al. [12] who found that Gal-3 (*LGALS-3* rs4652) gene variant was associated with increased galectin-3 expression levels.

Moreover, we found that the circulating IL-6 levels were highly elevated among type two diabetes with CAD than type two diabetes with no CAD than the control group and also statistically significantly higher among the genotypes C allele carriers (AC+CC). Remarkably, *Gal-3* expression levels among the carriers of the C allele containing genotypes (AC+CC) were positively and highly associated with IL-6 circulating levels.

High IL-6 levels are related to high body inflammatory state and adverse effects for multiple disease processes, such as unstable angina and septic shock [31]. Similarly, it is involved in the pathogenesis and progress of T2DM and related cardiovascular complications [32].

Alturfan et al. found that the *Gal-3* levels were positively and significantly correlated with plasma levels of IL-6 in the control and patients with AMI [33]. Also, Dong et al. [34] showed that *Gal-3* knockout by *Gal-3* RNA interference (si-RNA) significantly decreases the levels of the pro-inflammatory cytokines, like IL-1, IL-6, and NF-kb.

Modified lipoproteins can induce macrophage expression of *Gal-3*, and it was shown in atherosclerotic lesions in foam cells and macrophages [35]. *Gal-3* expression was specially upgraded in the dysfunctional regions of human atherosclerotic plaques, and the local *Gal-3* concentration increase in atherosclerotic lesions increased the pro-inflammatory state by enhancing the reclamation of monocytes and macrophages on the artery [36]. *Gal-3* can also en courage the activation in atherosclerotic plaques of vascular smooth muscle cells induced by oxidised LDL [8].

However, the findings of the relationship between atherosclerosis and Gal-3 were incoherent. A trial studied apolipoprotein E-deficient mouse and inactivation of the *Gal-3* gene showed decreasing atherosclerotic lesions and lower inflammation of low active *Gal-3* animals [37].

Nevertheless, Iacobini et al. suggested that the removal of *Gal-3* increased atherosclerosis degree in mice fed high-fat diets [38].

The association between the elevated levels of *Gal-3* and increased intimate thickness of carotids was established; high levels of *Gal-3* were also correlated with an augmented risk of CVD mortality in peripheral artery disease patients [26].

However, the previous studies showed that *Gal-3* levels in diabetes type two and metabolic syndrome are higher, the effect of *Gal-3* is contradictory in diabetic patients [39].

Menini et al. [9] has shown *Gal-3*'s significance in initiating and advancing T2DM long-term complications because of its capacity to link both advanced glycation end products (AGEs) and advanced lip-oxidation end products (ALEs) that accumulate in the target organ and exert toxic effects by activating proinflammatory and pro-oxidant pathways. However, Iacobini et al. [40] found that *Gal-3* had a role in protection against diabetic nephropathy and was thought to have a direct anti-inflammatory effect because of its AGEs plus ALEs receptor function.

Regarding the genome-wide association study (GWAS), there are plentiful single-nucleotide variants in the LGALS-3 gene that affect its gene expression; the lead SNP rs2274273 of the LGALS-3 locus that lies in high LD with two non-synonymous alternatives (rs4644; r^2^=1.0 and rs4652; r^2^=0.91) [30].

Unlike our observations, a report by Zhang et al. [41] showed that both SNPs rs2274273 and rs4644 gene variants of *LGALS-3* were associated with cardiovascular diseases in the Chinese population.

Djordjevic et al. [42] found that SNP rs2274273 gene variant was associated with *LGALS-3* mRNA levels of expression in atherosclerosis tissue of human carotid plaque.

Also, Djordjevic et al. [13] found that rs2274273 SNP was significantly associated with relative expression levels of *LGALS-3* with left-ventricular maladaptive remodelling and HF occurring six months after MI. In contrast to wild type homozygote (CC), the carrier of the T allele with genotypes (CT+TT) had significantly higher relative *LGALS-3* mRNA.

Despite the relation between *Gal-3* and cardiovascular events in CAD patients [43] [44], combination data between *Gal-3* (*LGALS-3* rs4652) gene variant and its gene expression levels with the risk of CAD in T2DM patients among an Egyptian population have not been published to date.

The present study has some limitations that need to be acknowledged; firstly, the sample size was relatively minor, and lonely Egyptian subjects were included in this research. This research, therefore, needs to be argued further in future analysis with greater sample size and different ethnical population. Secondly, this study didn't assess the expression of the *LGALS-3* gene in tissues, therefore, it was not possible to withdraw from this study that significant rs4652 in the *LGALS-3* gene affected its heart tissue expression and were directly involved in fibrosis, inflammation, and atherosclerosis. Thirdly, we investigated only one SNP (rs4652) in the vicinity of locus *LGALS-3*. This may not be enough to assess the overall effect of the *LGALS-3* containing haplotype block. Further studies should be carried out in combination with CAD in T2DM on tag SNPs that cover the genotypic variance of this haplotype block.

Also, additional studies with larger sample sizes are required to investigate the interaction between genetic variant, gene expression, and circulating Galectin-3 in T2DM and cardiovascular diseases.

## Conclusion

We found a significant relationship between the SNP rs4652 variant in the *LGALS-3* gene of Galectin-3 and CAD risk in T2DM Egyptian patients. The amount of LGALS-3 gene expression is also related to SNP rs4652 variant in Egyptian patients with T2DM and CAD complications. So, Galectin-3 can be used as a new biomarker for atherosclerosis risk assessment helping to identify diabetic patients who may need an early CAD intervention to reduce the mortality of the cardiovascular disease and increase life expectancy. Also, Galectin-3 inhibitors may be a pos-sible therapy for atherosclerosis and cardiovascular obstacles of T2DM in the future.

## Ethical approval (including reference number)

The Ethical Board of the University of Zagazig, Faculty of Medicine, accepted this research (Zu-IRB# 5576-9-19).

*Acknowledgements. *We acknowledged all individuals included in our research.

## Funding

No funding source.

## Conflict of interest statement

All the authors declare that they have no conflict of interest in this work.
